# A randomized, double blind, placebo-controlled pilot study to assess the efficacy of erenumab in individuals with temporomandibular disorder

**DOI:** 10.22514/jofph.2025.027

**Published:** 2025-06-12

**Authors:** Harold C. Avila, Kurt Kroenke, George J. Eckert, Ana G. Gossweiler, Lorena del Carmen Galvez, Domenick T. Zero

**Affiliations:** ^1^Department of Orthodontics, Indiana University School of Dentistry, Indianapolis, IN 46202, USA; ^2^Department of Medicine, Indiana University School of Medicine, Indianapolis, IN 46202, USA; ^3^Regenstrief Institute, Indianapolis, IN 46202, USA; ^4^Department of Biostatistics and Health Data Science, Indiana University School of Medicine, Indianapolis, IN 46202, USA; ^5^Oral Health Research Institute, Indiana University School of Dentistry, Indianapolis, IN 46202, USA

**Keywords:** Temporomandibular disorder, Erenumab, Clinical trial, Myalgia, Pain

## Abstract

**Background**: Erenumab has proven efficacious in treating migraine headache. Temporomandibular disorder (TMD) is a painful disorder which has high co-occurrence rates with migraine. We hypothesized that erenumab-aooe may also be beneficial in reducing pain in TMD-related myalgia. **Methods**: This phase II randomized placebo-controlled clinical trial evaluated the safety and efficacy of the off-label use of erenumab in reducing TMD-related pain. The TMD diagnosis was established using the Diagnostic Criteria for Temporomandibular Disorders. The primary outcome was pain interference as assessed by the 0-to-10-point Brief Pain Inventory (BPI). Secondary outcomes were depression, anxiety and somatic symptoms; jaw function; and percent of days taking pain medication. Subjects were randomized at baseline to receive either erenumab 140 mg or placebo administered subcutaneously every 4 weeks for a total of five treatments. Outcome assessments were conducted at baseline, 4, 8, 12, 16, 20 and 24 weeks. **Results**: Thirty subjects were enrolled with 15 randomized to each treatment group. Baseline pain was mild (BPI interference of 2.19; BPI severity of 2.95). There were no significant treatment effects at any time points with the between-group BPI interference at 24 weeks being −0.19 (95% confidence interval −1.94 to 1.56; *p* = 0.82). The outcomes were similar between erenumab and placebo for all outcomes except the Patient Health Questionnaire 4-item scale (PHQ-4) which showed that depression/anxiety symptoms were modestly worse (*p* = 0.03) in the erenumab group. Five participants withdrew during the trial (4 in erenumab arm, 1 in placebo arm). **Conclusions**: Erenumab was not efficacious in reducing TMD myalgic pain in this phase II trial of 30 subjects with relatively mild pain. **Clinical Trial Registration**: The study was registered in clinicaltrials.gov (ID: NCT04884763).

## 1. Introduction

Aimovig® (erenumab-aooe) is a first in class Food and Drug Administration (FDA)-approved human monoclonal antibody for the prevention of migraine in adults. It selectively targets and blocks the calcitonin gene-related peptide (CGRP) receptor, disrupting a key component of migraine pathophysiology [1]. Several studies have provided evidence of the safety and efficacy of erenumab in reducing the frequency of migraine compared to placebo [2, 3]. Furthermore, an open-label longer-term study found that erenumab was safe and well-tolerated with a safety profile consistent with shorter-term placebo-controlled studies through 5-years of treatment [4]. Erenumab has rapidly become a widely accepted prescription drug for the prevention of migraine, including episodic migraine and chronic migraine along with other anti-CGRP monoclonal antibody-based therapies [5, 6].

Temporomandibular disorder (TMD) is known to be co-morbid with the medical diagnosis of chronic migraine [7, 8]. Temporomandibular disorder (TMD) is a common condition that may affect up to a third of the general population [9]. TMD is the most common orofacial pain condition of non-dental origin [10]. Additionally, TMD has a major adverse impact on health-related quality of life [11, 12] as well as health care costs [13]. There is increasing interest in the concept of Chronic Overlapping Pain Conditions (COPCs), which include TMD, fibromyalgia, irritable bowel syndrome, vulvodynia, chronic fatigue syndrome, interstitial cystitis/painful bladder syndrome, endometriosis, chronic tension-type headache, migraine headache, and chronic lower back pain that may have increased pain sensitivity as well as common genetic and biopsychosocial factors [14].

During 2018–2019, shortly after the commercial release of erenumab, one of the authors (HCA) used erenumab to treat 5 patients with chronic severe TMD pain and a history of migraine headaches. Four of these patients had substantial reductions in pain following this off-label administration of erenumab. These promising treatment results were the impetus for the pilot trial reported in this paper.

Chronic migraine is thought to originate within the trigeminovascular pathway (TGV) [15, 16]. TMD is also considered to originate within the TGV [17]. Thus, our working hypothesis is that a CGRP receptor antagonist for treatment of chronic migraine will also be effective in reducing TMD pain and related symptoms. The purpose of this proof-of-concept study was to evaluate the safety and efficacy of erenumab in reducing Temporomandibular Disorder (TMD) pain compared to placebo. The study design was a phase II randomized placebo-controlled clinical trial. We postulated that erenumab would be superior to placebo in reducing pain intensity/severity over 20 weeks. Secondary outcomes included depression, anxiety and somatic symptoms; jaw function; and percentage of days taking pain medication.

## 2. Methods

### 2.1 Study participants

#### 2.1.1 Inclusion criteria

Eligible participants included adults (age 18 to 59 years) who were diagnosed as having pain-related TMD using the diagnostic criteria (DC/TMD) for “myalgia”, recommended by the International RDC/TMD Consortium Network and Orofacial Pain Special Interest Group [18]. Eligible participants also had to have a history of head, face, neck, and/or shoulder pain for longer than 3 months; a good knowledge of the English language; and if taking prescription pain medication, a stable dose regiment for at least 2 months prior to the screening visit.

The Diagnostic Criteria for Temporomandibular Disorders Symptom Questionnaire and DC/TMD Examination Form was used to confirm the TMD diagnosis [19]. To meet the diagnostic criteria for TMD Myalgia (IDC-9 729; ICD-10 M79.1), subjects must have had a history of pain of muscle origin that was affected by jaw movement, function or parafunction, and demonstrated replication of this pain with provocation testing of the masticatory muscles [18]. No minimum pain threshold was used for eligibility because the DC/TMD criteria require documentation of pain but no specific level of pain severity. The criteria included having a positive history for both pain in the jaw, temple, in the ear, or in front of ear and pain modified with jaw movement, function or parafunction. During clinical examination, subjects must have had a confirmation of pain in the temporalis or masseter muscle(s) and report familiar pain in the temporalis or masseter muscle(s) with at least one of the following provocation tests: palpation of the temporalis or masseter muscle(s) or maximum unassisted or assisted opening movement(s).

#### 2.1.2 Exclusion criteria

Subjects that met any of the following exclusion criteria were not eligible: (1) lacking stable bilateral posterior occlusion; (2) currently using a complete maxillary or mandibular prosthetic denture; (3) currently undergoing TMD treatment elsewhere (exception is subjects undergoing TMD treatment involving the use of oral orthotics for a minimum of 3 months prior to screening can be considered eligible for the study); (4) started orthodontic treatment during the 3 months prior to Screening; (5) currently included in other experimental protocols within the last 30 days or 5 half-lives before enrollment; (6) currently pregnant, planning to become pregnant or breastfeeding; (7) allergic to erenumab or any of the ingredients in Aimovig® (acetate, polysorbate 80 and sucrose); (8) allergic to rubber or latex; (9) having 8 or more migraine days during the past 4 weeks; (10) started receiving massage, acupuncture or physical therapy treatment of the head, neck or shoulders during the previous 3 months prior to Screening; (11) history of unstable or acute severe non-head, neck or shoulder pain; (12) history of traumatic brain injury; (13) history of surgical treatment or recommended surgical treatment for TMD; (14) history of ongoing, unresolved disability litigation; (15) history of drug abuse; (16) started treatment for moderate to severe sleep apnea requiring continuous positive airway pressure (CPAP) or oral mandibular repositioning appliance during the previous 3 months prior to Screening; (17) history of previously receiving erenumab-aooe or other anti-CGRP pathway therapies, including anti-CGRP pathway treatments; (18) history of chronic constipation and/or using medication associated with decreased gastrointestinal motility; (19) history of uncontrolled hypertension or risk factors for hypertension; (20) anything that would place the subject at increased risk or preclude the individual’s full compliance with or completion of the study (*e.g.*, medical condition, laboratory finding, physical exam finding logistical complication).

#### 2.1.3 Enrollment procedures

Participants were recruited from November 2021 through July 2023 using fliers and advertisements placed in the Indiana University (IU) School of Dentistry (IUSD) and other locations on the Indiana University Purdue University Indianapolis (IUPUI) campus including IU Health facilities. We also used social media advertisements. Persons responding to an advertisement were given a brief description of the study and asked a series of questions related to the inclusion/exclusion criteria using an institutional review board (IRB) approved phone script. Those who were and appeared to meet the study requirements were scheduled for a screening visit at the Oral Health Research Institute (OHRI). A Study Dentist qualified to diagnosis TMD reviewed the potential subject’s health history, medications and TMD history for the inclusion and exclusion criteria.

### 2.2 Treatment

#### 2.2.1 Treatment arms

Patients were randomized at baseline to one of the two treatment arms:

● Arm A: erenumab 140 mg subcutaneous, administered every four weeks for a total of five treatments.

● Arm B: placebo subcutaneous, administered every four weeks for a total of five treatments.

Randomization was stratified based on sex into two groups using block randomization based on a schedule provided by the study statistician. Subjects, investigators, and study staff remained blinded to the identity of the treatment from the time of randomization until database lock. The randomization code was kept strictly confidential, and the identity of the study drug treatments concealed using identical packaging and labeling.

#### 2.2.2 Dispensing of study drug

The study sponsor provided the active and placebo free of charge through the Investigator Sponsored Studies (ISS) Program (CAMG334AUS01T). The investigational products (erenumab and placebo) were supplied in prefilled syringes, using identical packaging and labeling and shipped through ISS to the unblinded IU Health Investigational Drug Services Pharmacy. The IU Health Pharmacy dispensed the investigational products according to a randomization schedule provided by the study statistician. Doses were administered in the upper arm, thigh, or abdomen by a study dentist qualified in subcutaneous drug administration.

Study products were stored and handled according to labeling instructions and stored in a secure area of the IU Health Investigation Drug Services Pharmacy to which only the pharmacy staff had access. The IU Health Pharmacy maintained records documenting the receipt, use, loss or other disposition of the products on the electronic Investigational Agent Accountability Record. The clinical site, OHRI, working with the pharmacy also maintained a Drug Administration Form documenting the date and time of transport to the blinded site staff, the date and signature of the blinded site staff receiving the study drug, the date and time the study drug was received from the pharmacy, the date and time the study drug was administered, and the randomized injection site body location noted by the study dentist. These procedures coupled with the use of identical prefilled syringes for the erenumab, and placebo groups assured blinding of the study subjects, investigators, research staff and outcome assessors.

### 2.3 Study outcomes

At Baseline and Weeks 4, 8, 12, 16, 20 and 24 subjects were instructed to complete patient-reported outcomes regarding pain and other TMD-relevant symptoms. The outcome measures were based on consensus recommendations for research assessments in chronic pain and TMD research [19, 20].

The primary study outcome was the Brief Pain Inventory (BPI) pain severity scale which rates the severity of pain on 4 items (current, worst, least and average pain in past week) [21, 22, 23]. Each item is rated on a 0 (no pain) to 10 (pain as bad as you can image) scale. The BPI pain severity score is the average of the items and ranges from 0 to 10, with higher scores representing greater pain interference.

Three other pain outcomes were assessed. The Brief Pain Inventory (BPI) pain interference scale rates pain-related interference in 7 areas (mood, physical activity, work, social activity, relations with others, sleep, and enjoyment of life). Each item is rated on a 0 (does not interfere) to 10 (completely interference) scale [21]. The BPI pain interference score is the average of the items and ranges from 0 to 10, with higher scores representing greater pain interference. The Patient Global Impression of Change (PGIC) assesses change in pain on a 7-item Likert scale where 1 = much better; 2 = moderately better; 3 = a little better; 4 = no change; 5 = a little worse; 6 = moderately worse; 7 = much worse [24]. Daily use of pain medications was tracked each month by asking how many days medications were taken for TMD-related pain.

Depressive and anxiety symptoms were assessed by the Patient Health Questionnaire (PHQ-4) which comprises 2 depression items and 2 anxiety items [25, 26, 27]. Individuals are asked how much they have been bothered by each of the symptoms during the past 2 weeks on a scale of 0 (not at all) to 3 (nearly every day). The PHQ-4 total score ranges from 0 to 12 with higher scores representing more severe symptoms. Jaw function was assessed with the Jaw Function Limitation Scale (JFLS-8) which asks the level of limitation during the past month in 8 activities (chew tough food; chew chicken; eat soft food requiring no chewing; open wide enough to drink from a cup; swallow; yawn; talk; smile) [28, 29]. Each item is scored from 0 (no limitation) to 10 (severe limitation). The JFLS-8 score is the average of the 8 items and ranges from 0 to 10, with higher scores representing greater jaw functional impairment. The Somatic Symptom Scale (SSS-8) asks how much each of 8 common physical symptoms have bothered the individual during the past 7 days on a 5-point Likert scale ranging from 0 (not at all) to 4 (very much) [30]. Total scores range from 0 to 32 with higher scores representing higher somatic symptom burden.

### 2.4 Safety monitoring

Adverse events were assessed and documented at each follow-up visit. This study was conducted in compliance with the US Code of Federal Regulations (CRF) governing informed consent, the IRB, and Investigator conduct. This study was performed according to Good Clinical Practice for research. Standard operating procedures for the trial were on file with the Quality Assurance staff of the Oral Health Research Institute. All study staff who had direct contact with subjects were required to review the WARNINGS AND PRECAUTIONS for Hypersensitivity Reactions, Constipation with Serious Complications and Hypertension found in Section 5 of the US Prescribing Information (USPI) for Aimovig.

### 2.5 Statistical analysis

A sample size of 12 subjects per group has been suggested for pilot studies to evaluate feasibility and to estimate group means and standard deviations (SD) for future study planning [31, 32]. Based on two-sided paired *t*-tests and two-sample *t*-tests, all conducted at a 5% significance level, this pilot study had 80% power to detect effect sizes of 0.9 for changes over time within groups and effect sizes of 1.2 for differences between groups. To account for dropout, the study enrolled 15 subjects per group, for a total of 30 subjects.

Mixed model repeated measures (MMRM) analysis was used to evaluate changes over time in the BPI pain and BPI interference scores, JFLS-8, PHQ-4 total score, PHQ-4 anxiety, and depression scores, SSS-8 Scale, and PGIC pain change within and between treatment groups. The MMRM included factors for treatment group, time, and their interaction. The MMRM also included sex as a covariate due to stratification by sex in the randomization. A two-sided 5% significance level was used for all tests without multiplicity adjustment between multiple endpoints.

## 3. Results

### 3.1 Subject enrollment

Thirty-two individuals met the inclusion/exclusion criteria. Two individuals who qualified for the study were never randomized to study treatment; one decided not to continue and the other due to scheduling issues. A total of 30 subjects, 26 females and 4 males (equally balanced between erenumab and Placebo groups), median age of 34 years old (range 21 to 58 years old) were randomized into the study. Table 1 summarizes demographic characteristics of the sample. The mean BPI interference and severity scores were in the mild range (2.19 and 2.95, respectively).

**Table 1.** Baseline characteristics of study participants.

Characteristic		Total Sample (n = 30)	Erenumab Arm (n = 15)	Placebo Arm (n = 15)
Age, mean (SD)		34.8 (8.6)	34.9 (8.9)	34.7 (8.5)
Sex, n				
	Female	26	13	13
	Male	4	2	2
Race, n				
	White	28	14	14
	Black	2	1	1
Ethnicity, n				
	Not Hispanic	22	10	12
	Hispanic	8	5	3

*SD: standard deviations.*

Twenty-two subjects completed the study (10 Erenumab; 12 Placebo). Fig. 1 shows the participant flow in this randomized trial. Four subjects in the erenumab group withdrew from the study for the following reasons: constipation, which was considered by the PI to be definitely associated with the investigational product; concern about hypertension; family issues; and desire to donate plasma. One subject in the Placebo group withdrew from the study because the treatment was not improving pain. Three subjects were lost to follow-up after repeated attempts to contact them. There were no gender differences between treatment groups and no differences in the percentage of subjects seen at each follow-up visit between groups.

**Fig. 1.** Participant flow in the randomized trial.

### 3.2 Efficacy outcomes

The study findings are presented for the primary outcome of pain interference and the secondary pain outcomes of pain severity and global change in pain in Fig. 2. There were no significant differences in pain outcomes between treatment arms. In the small subset of 7 subjects (4 on erenumab, 3 on placebo) who had more than mild pain (*i.e.*, BPI ≥4), pain outcomes were similar. Table 2 summarizes findings for all study outcomes. Overall, the outcomes were similar between erenumab and placebo for all outcomes except the PHQ-4 which showed that depression/anxiety symptoms were modestly worse in the erenumab group. Although between-group PHQ-4 differences were not significant at all 7 timepoints, the overall effect using repeated measures analysis was significant (*p* = 0.032).

**Fig. 2.** Mean (adjusted for sex) Brief Pain Inventory (BPI) Interference and Severity scores and Patient Global Impression of Change (PGIC) scores with 95% CI by treatment group.

**Table 2.** Outcome comparisons by treatment arm (adjusted for sex)*.

Outcome (scale range)	Week	Erenumab Mean (95% CI)	Placebo Mean (95% CI)	Difference Mean (95% CI)	*p*-value
BPI interference (0–10)					
	0	2.2 (1.2, 3.2)	2.0 (1.0, 2.9)	0.2 (−1.1, 1.6)	0.71
	4	1.3 (0.4, 2.2)	1.3 (0.5, 2.2)	0.0 (−1.2, 1.1)	0.96
	8	1.1 (0.3, 2.0)	1.2 (0.5, 2.0)	−0.1 (−1.1, 0.9)	0.86
	12	0.9 (0.3, 1.4)	0.9 (0.4, 1.4)	0.0 (−0.7, 0.6)	0.99
	16	1.2 (0.2, 2.3)	1.4 (0.4, 2.4)	−0.2 (−1.5, 1.2)	0.79
	20	1.1 (0.3, 1.9)	1.0 (0.2, 1.7)	0.2 (−0.9, 1.2)	0.78
	24	1.2 (−0.1, 2.5)	1.4 (0.2, 2.7)	−0.2 (−1.9, 1.6)	0.82
BPI severity (0–10)					
	0	2.7 (1.7, 3.7)	2.6 (1.6, 3.6)	0.1 (−1.2, 1.4)	0.87
	4	2.0 (0.9, 3.1)	1.7 (0.7, 2.8)	0.3 (−1.1, 1.6)	0.71
	8	1.7 (0.7, 2.7)	1.7 (0.8, 2.7)	−0.1 (−1.2, 1.1)	0.93
	12	2.0 (1.1, 3.0)	1.3 (0.4, 2.1)	0.8 (−0.3, 1.8)	0.14
	16	2.1 (1.0, 3.2)	1.7 (0.7, 2.8)	0.3 (−1.0, 1.7)	0.60
	20	1.7 (0.8, 2.7)	1.4 (0.6, 2.3)	0.3 (−0.8, 1.4)	0.57
	24	2.0 (0.9, 3.1)	1.5 (0.5, 2.6)	0.5 (−0.9, 1.8)	0.47
Jaw function limitation scale (0–10)					
	0	1.7 (0.9, 2.4)	1.8 (1.0, 2.5)	−0.1 (−1.1, 0.9)	0.80
	4	1.0 (0.3, 1.8)	1.3 (0.5, 2.0)	−0.2 (−1.2, 0.8)	0.64
	8	0.8 (0.2, 1.4)	1.1 (0.5, 1.7)	−0.3 (−1.1, 0.5)	0.43
	12	0.8 (0.2, 1.3)	0.7 (0.2, 1.2)	0.1 (−0.6, 0.7)	0.82
	16	1.0 (0.2, 1.8)	1.0 (0.3, 1.7)	0.0 (−1.0, 1.0)	0.97
	20	0.5 (−0.2, 1.7)	0.5 (−0.1, 1.1)	0.0 (−0.8, 0.8)	0.97
	24	0.9 (0.2, 1.7)	1.2 (0.5, 1.9)	−0.2 (−1.2, 0.7)	0.61
PHQ-4 (0–12)					
	0	2.6 (1.2, 3.9)	2.0 (0.7, 3.4)	0.5 (−1.1, 2.2)	0.51
	4	3.4 (1.8, 5.1)	1.5 (0.0, 3.1)	1.9 (−0.1, 3.9)	0.06
	8	2.9 (1.6, 4.2)	1.1 (−0.1, 2.4)	1.8 (0.2, 3.3)	0.03
	12	4.2 (2.0, 6.3)	1.8 (−0.2, 3.8)	2.4 (−0.4, 5.2)	0.09
	16	2.9 (1.2, 4.5)	1.7 (0.1, 3.2)	1.2 (−0.9, 3.3)	0.24
	20	4.4 (2.4, 6.5)	1.6 (−0.3, 3.5)	2.8 (0.2, 5.4)	0.04
	24	3.6 (2.2, 5.0)	1.0 (−0.3, 2.3)	2.6 (0.9, 4.2)	0.003
SSS-8 (0–32)					
	0	6.2 (3.4, 8.9)	8.0 (5.2, 10.7)	−1.8 (−5.2, 1.6)	0.28
	4	7.2 (4.1, 10.2)	5.4 (2.5, 8.2)	1.8 (−1.9, 5.4)	0.33
	8	6.2 (3.7, 8.7)	4.5 (2.1, 6.8)	1.8 (−1.0, 4.5)	0.21
	12	7.8 (4.9, 10.7)	5.4 (2.7, 8.0)	2.5 (−0.9, 5.9)	0.14
	16	6.8 (3.7, 9.9)	7.5 (4.6, 10.4)	−0.7 (−4.4, 3.1)	0.71
	20	6.5 (3.3, 10.0)	6.7 (3.9, 9.6)	−0.3 (−4.0, 3.4)	0.89
	24	6.7 (3.5, 9.9)	5.5 (2.5, 8.5)	1.2 (−2.7, 5.1)	0.54
Patient global impression of change (1–7)					
	4	3.5 (2.8, 4.2)	3.7 (3.1, 4.4)	−0.3 (−1.0, 0.5)	0.46
	8	3.7 (2.9, 4.5)	3.9 (3.1, 4.6)	−0.2 (−1.1, 0.8)	0.73
	12	3.5 (2.6, 4.4)	3.6 (2.8, 4.3)	−0.1 (−1.1, 1.0)	0.92
	16	3.5 (2.7, 4.4)	3.8 (3.0, 4.6)	−0.3 (−1.3, 0.7)	0.58
	20	3.5 (2.6, 4.4)	3.6 (2.8, 4.4)	−0.1 (−1.1, 1.0)	0.91
	24	3.7 (2.9, 4.5)	3.6 (2.8, 4.3)	0.1 (−0.8, 1.0)	0.76
Medicine for jaw pain % d/mon					
	4	18.6 (−0.3, 37.4)	10.1 (−6.6, 26.8)	10.9 (−3.8, 25.7)	0.14
	8	11.2 (−7.1, 29.6)	9.1 (−7.2, 25.4)	5.6 (−10.9, 22.2)	0.49
	12	12.0 (−6.7, 30.6)	9.4 (−7.2, 25.9)	7.6 (−11.3, 26.5)	0.41
	16	13.1 (−7.2, 33.4)	9.8 (−8.4, 27.9)	1.2 (−17.7, 20.0)	0.90
	20	23.0 (0.8, 45.1)	5.4 (−14.4, 25.2)	12.1 (−9.5, 33.7)	0.26
	24	13.7 (−5.2, 32.6)	6.6 (−10.2, 23.5)	6.0 (−11.7, 23.7)	0.49

*Higher score is worse for all scales. Difference = placebo minus erenumab score. BPI: Brief Pain Inventory; CI: confidence interval; PHQ: Patient Health Questionnaire; SSS: Somatic Symptom Scale.

### 3.3 Safety evaluations

Eleven potential study-related adverse events (AEs) were reported in 6 subjects (5 in erenumab arm and 1 in placebo arm). These 11 AEs included irritation at injection site (2 erenumab, 2 placebo), myalgia (1 erenumab,1 placebo), nausea (2 erenumab), constipation (1 erenumab), drowsiness (1 erenumab) and COVID-19 symptoms (1 placebo).

## 4. Discussion

The findings of this randomized controlled pilot study do not support the premise that erenumab is beneficial in reducing facial, jaw or TMD pain intensity/severity in individuals with pain using the diagnostic criteria (DC/TMD) for “myalgia”. This finding was consistent for the primary outcome measure pain interference using the Brief Pain Inventory (BPI) as well the secondary pain-related measures BPI pain severity, global improvement in pain (PGIC), jaw function limitation (JFLS-8), somatic symptom severity (SSS-8), and days of use of TMD pain-specific medication per month.

There are several possible reasons for the lack of benefit of erenumab compared to placebo for pain. Firstly, study participants had only relatively low levels of pain at baseline; both groups had a mean pain score <3 which, on a 0 to 10 numeric rating scale, indicates mild pain [33]. This could have created a floor effect in our study’s ability to show a reduction in pain. It is possible that erenumab’s separation from placebo might differ in patients with more severe TMD myalgic pain. Notably, all secondary scales had relatively low scores at baseline suggesting a sample with mild overall symptoms and good jaw function. Secondly, the placebo response may be particularly high in some TMD patients and, in a small sample, might have contributed to our null findings [34]. Thirdly, it may be that our hypothesis was incorrect that erenumab would be beneficial in TMD pain because of its comorbidity with migraine and potential shared pathways.

Unexpectantly, there were worse results for erenumab compared to placebo for depression and anxiety based on the Patient Health Questionnaire (PHQ-4). This isolated finding should be put in the context of what is known about the psychological effects of erenumab in previous trials for migraine as well as post-marketing data. Firstly, the severity of depression and anxiety symptoms was relatively low. PHQ-4 scores of 3–5 are considered mild [25], and scores were <3 in both groups at baseline and never rose above 4.4 in the erenumab group at any assessment. Secondly, the between-group differences at most follow-up time points were only mildly statistically significant which is important because multiple secondary outcomes were tested. Thus, it is possible the single secondary outcome differing between groups represents a chance finding. Thirdly, data regarding the psychological effects of erenumab are inconclusive. Data from trials have not found it to be a common adverse event, and there is some evidence from other studies that erenumab might even be beneficial for depression and anxiety [35]. In the post-marketing setting, there is a slight increase in the reporting of depression and anxiety with erenumab compared to other acute or preventive migraine treatments [36]. It should be noted, however, that findings from disproportionality analyses do not confirm causality. Importantly, 3 systematic reviews have not found psychological symptoms to differ between erenumab and placebo [37, 38, 39]. Thus, a large body of evidence including Phase III trial data coupled with extensive post-marketing surveillance do not indicate that erenumab has psychoactive effects.

The most important study limitation is the generally mild level of pain and other secondary outcomes at baseline which reduced the amount of improvement that could be detected (*i.e.*, a floor effect). Second, the primary outcome was assessed with one of the most commonly recommended general pain measures. Measuring TMD-specific pain may have also been informative.

## 5. Conclusions

In conclusion, erenumab compared to placebo was not effective in reducing pain in a small pilot trial of patients with TMD with low pain intensity. Whether the medication might be beneficial in patients with more severe pain requires further research. For now, the use of erenumab in treating TMD-related pain in the absence of comorbid chronic migraine cannot be recommended.
